# 4-(Dec­yloxy)phenyl 2-oxo-7-trifluoro­methyl-2*H*-chromene-3-carboxyl­ate

**DOI:** 10.1107/S1600536813008222

**Published:** 2013-03-28

**Authors:** B. S. Palakshamurthy, H. C. Devarajegowda, H. T. Srinivasa, S. Sreenivasa, Vijithkumar

**Affiliations:** a Department of Physics, Yuvaraja’s College (Constituent College), University of Mysore, Mysore, Karnataka 570 005, India; b Raman Research Institute, C. V. Raman Avenue, Sadashivanagar, Bangalore, Karnataka, India; c Center for Advanced Materials and Department of Chemistry, Tumkur University, Tumkur, Karnataka 572103, India; d Solid State and Structural Chemistry Unit, Indian Institute of Science, Bangalore 560 012, India

## Abstract

The title compound, C_27_H_29_F_3_O_5_, is a liquid crystal (LC) and exhibits enanti­otropic SmA phase transitions. In the crystal, the dihedral angle between the 2*H*-chromene ring system and the benzene ring is 62.97 (2)°. The three F atoms of the –CF_3_ group are disordered over two sets of sites with occupancy factors 0.71 (4):0.29 (4). In the crystal, pairs of C—H⋯O hydrogen bonds form inversion dimers and generate *R*_2_^2^(10) rings. The structure also features C—H⋯F and C—H⋯π inter­actions along [100] and [010], respectively.

## Related literature

For the synthesis and liquid crystal behaviour of the title compound, see: Mahadevan *et al.* (2013 ▶). For the biological activity of coumarins and their derivatives, see: Borges *et al.* (2005 ▶); Kontogiorgis & Hadjipavlou-Litina (2005 ▶) and for their industrial applications, see: Hejchman *et al.* (2011 ▶). For the structure of 4-(oct­yloxy)phenyl 2-oxo-2*H*-chromene-3-carboxyl­ate, see: Palakshamurthy *et al.* (2013 ▶). For hydrogen-bond motifs, see: Bernstein *et al.* (1995 ▶).
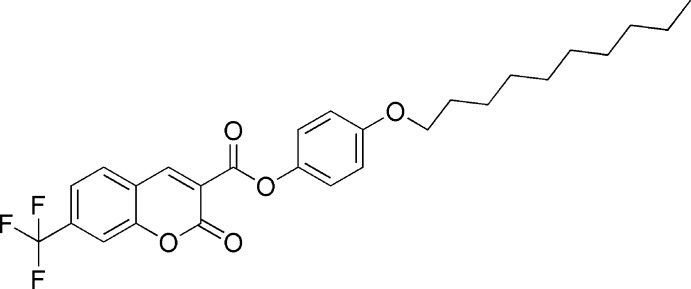


## Experimental

### 

#### Crystal data

C_27_H_29_F_3_O_5_
*M* *_r_* = 490.50
Monoclinic, 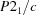

*a* = 27.85 (3) Å
*b* = 9.281 (10) Å
*c* = 9.981 (11) Å
β = 94.849 (18)°
*V* = 2571 (5) Å^3^
*Z* = 4
Mo *K*α radiation
μ = 0.10 mm^−1^
*T* = 99 K
0.52 × 0.42 × 0.40 mm

#### Data collection

Bruker APEXII diffractometer
Absorption correction: multi-scan (*SADABS*; Sheldrick, 2007 ▶) *T*_min_ = 0.950, *T*_max_ = 0.961
21163 measured reflections
4448 independent reflections
2547 reflections with *I* > 2σ(*I*)
*R*_int_ = 0.073

#### Refinement

*R*[*F* ^2^ > 2σ(*F* ^2^)] = 0.084
*wR*(*F* ^2^) = 0.221
*S* = 1.07
4448 reflections
344 parameters
H-atom parameters constrained
Δρ_max_ = 0.18 e Å^−3^
Δρ_min_ = −0.17 e Å^−3^

### 

Data collection: *APEX2* (Bruker, 2009 ▶); cell refinement: *APEX2* and *SAINT-Plus* (Bruker, 2009 ▶); data reduction: *SAINT-Plus* and *XPREP* (Bruker, 2009 ▶); program(s) used to solve structure: *SHELXS97* (Sheldrick, 2008 ▶); program(s) used to refine structure: *SHELXL97* (Sheldrick, 2008 ▶); molecular graphics: *ORTEP-3 for Windows* (Farrugia, 2012 ▶) and *Mercury* (Macrae *et al.*, 2008 ▶); software used to prepare material for publication: *SHELXL97*.

## Supplementary Material

Crystal structure: contains datablock(s) I, global. DOI: 10.1107/S1600536813008222/sj5309sup1.cif

Structure factors: contains datablock(s) I. DOI: 10.1107/S1600536813008222/sj5309Isup2.hkl

Supplementary material file. DOI: 10.1107/S1600536813008222/sj5309Isup3.cml

Additional supplementary materials:  crystallographic information; 3D view; checkCIF report

## Figures and Tables

**Table 1:** Hydrogen-bond geometry (Å, °) *Cg*1 and *Cg*2 are the centroids of the C2–C7 and C12–C17 rings respectively.

*D*—H⋯*A*	*D*—H	H⋯*A*	*D*⋯*A*	*D*—H⋯*A*
C10—H10⋯O3^i^	0.95	2.56	3.396 (5)	146
C27—H27*C*⋯F1^ii^	0.98	2.52	3.432 (13)	154
C6—H6⋯*Cg*2^i^	0.95	3.09	3.899	144
C16—H16⋯*Cg*2^iii^	0.95	3.33	4.120	142
C3—H3⋯*Cg*1^iv^	0.95	3.43	4.325	163

Symmetry codes: (i) 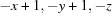; (ii) 
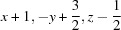
; (iii) 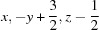; (iv) 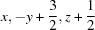.

**Fig. 1.**
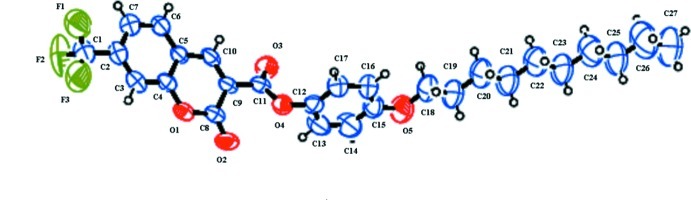
Molecular structure of the title compound, showing displacement ellipsoids drawn at the 50% probability level. Only the major component of the disordered CF3 group is shown.

**Fig. 2.**
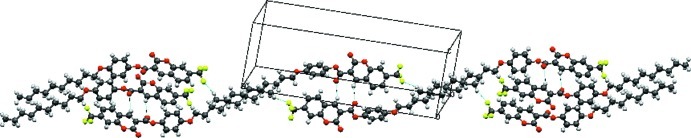
Crystal packing of the title compound with hydrogen bonds drawn as dashed lines.

**Fig. 3.**
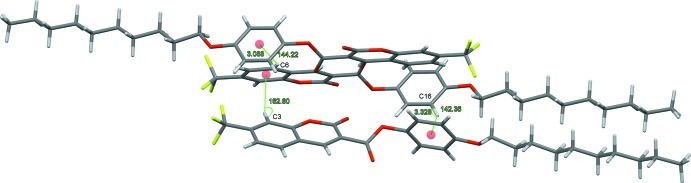
Packing of the title compound. C—H···π interactions are shown as dashed lines.

## supplementary crystallographic information

### Comment

The title compound is a liquid crystal (LC) exhibiting enantiotropic SmA phase transitions at 203.9(*22.64*) on heating and at 135.1(*47.57*) on cooling [The transition temperature in °C and the associated enthalpy values in kJ mol^-1^ (in italics)] (Mahadevan *et al.*, 2013).

Organic compounds with a 2*H*-chromene ring system and their derivatives display a wide range of biological activities such as antiviral (Borges *et al.*., 2005) and anti-inflammatory (Kontogiorgis *et al.*, 2005) activity. They also display photochemical and photophysical properties, acting as molecular fluorescent sensors, laser dyes and have many industrial applications (Hejchman *et al.*, 2011). Keeping this in mind we report here the structure of the 4-(decyloxy)phenyl 7-(trifluoromethyl)-2-oxo-2*H*-chromene-3-carboxylate(I), and its comparision with 4-(octyloxy)phenyl 2-oxo-2*H*-chromene-3 –carboxylate(II) (Palakshamurthy *et al.*, 2013).

The asymmetric unit of 4-(decyloxy)phenyl 7-(trifluoromethyl)-2-oxo-2*H*-chromene-3-carboxylate is shown in Fig.1. The three F atoms of the –CF_3_ group are disordered over two sets of sites with occupancy factors 0.71 (4):0.29 (4). The dihedral angles between the 2*H*-chromene ring and the benzene ring are 62.97 (2)^o^ and 21.11 (1)° in the compounds I and II respectively. The crystal structure is characterized by intermolecular C10—H10···O3 hydrogen bonds that form inversion dimers and generate a *R*^2^_2_(10) ring pattern (Bernstein *et al.*, 1995). C27—H27···F1 hydrogen bonds then link the dimers into chains along *a* . The structure is further stabilized by C3—H3···Cg1, C6—H6···Cg2 and C16—H16···Cg2 interactions, Table 1.

### Experimental

A mixture of 7-(trifluoromethyl)-2-oxo-2*H*-chromene-3-carboxylic acid (0.100 g, 0.1 mmol), 4-(decyloxy) phenol (0.100 g, 0.1 mmol) dicyclohexylcarbodiimide (DCC) (0.100 g, 0.1 mmol) and catalytic quantity of DMAP (*N*,*N*-dimethyl amino pyridine) were stirred at room temperature for 48hrs in dry dichloromethane. Progress of the reaction was monitored by TLC (ethyl acetate: pet ether 2:8). After the completion of the reaction, the reaction mass was diluted with water and extracted into dichloromethane (25 ml). The organic layer was washed with water and dried over anhydrous sodium sulfate. The crude product thus obtained was purified by column chromatography using ethyl acetate: petroleum ether (2:8) as eluent followed by recrystallization from ethanol. A single crystal suitable for X-ray diffraction was grown from ethanol.

### Refinement

The H atoms bound to carbon were positioned with idealized geometry using a riding model with d(C–H) = 0.93- 0.99 Å. All C–H atoms were refined with isotropic displacement parameters set to 1.2–1.5 *U*_eq_(C). The F1, F2, and F3 fluorine atoms of the –CF_3_ group were disordered over two sites and refined with site occupancy factors 0.71 (4):0.29 (4). The crystals were not of high quality which accounts for the high uncertainties in the lengths of the unit cell axes and the relatively high residuals.

### Crystal data

**Table:**

C_27_H_29_F_3_O_5_	prism
*M**_r_* = 490.50	*D*_x_ = 1.267 Mg m^−^^3^
Monoclinic, *P*2_1_/*c*	Melting point: 418 K
Hall symbol: -P 2ybc	Mo *K*α radiation, λ = 0.71073 Å
*a* = 27.85 (3) Å	Cell parameters from 2547 reflections
*b* = 9.281 (10) Å	θ = 2.2–25°
*c* = 9.981 (11) Å	µ = 0.10 mm^−^^1^
β = 94.849 (18)°	*T* = 99 K
*V* = 2571 (5) Å^3^	Prism, colourless
*Z* = 4	0.52 × 0.42 × 0.40 mm
*F*(000) = 1032	

### Data collection

**Table:**

Bruker APEXII diffractometer	4448 independent reflections
Radiation source: fine-focus sealed tube	2547 reflections with *I* > 2σ(*I*)
Graphite monochromator	*R*_int_ = 0.073
φ and ω scans	θ_max_ = 25.0°, θ_min_ = 2.2°
Absorption correction: multi-scan (*SADABS*; Sheldrick, 2007)	*h* = −33→33
*T*_min_ = 0.950, *T*_max_ = 0.961	*k* = −11→11
21163 measured reflections	*l* = −11→11

### Refinement

**Table:**

Refinement on *F*^2^	Primary atom site location: structure-invariant direct methods
Least-squares matrix: full	Secondary atom site location: difference Fourier map
*R*[*F*^2^ > 2σ(*F*^2^)] = 0.084	Hydrogen site location: inferred from neighbouring sites
*wR*(*F*^2^) = 0.221	H-atom parameters constrained
*S* = 1.07	*w* = 1/[σ^2^(*F*_o_^2^) + (0.0928*P*)^2^ + 0.7006*P*] where *P* = (*F*_o_^2^ + 2*F*_c_^2^)/3
4448 reflections	(Δ/σ)_max_ = 0.004
344 parameters	Δρ_max_ = 0.18 e Å^−^^3^
0 restraints	Δρ_min_ = −0.17 e Å^−^^3^
0 constraints	

### Special details

**Table:**

Geometry. All e.s.d.'s (except the e.s.d. in the dihedral angle between two l.s. planes) are estimated using the full covariance matrix. The cell e.s.d.'s are taken into account individually in the estimation of e.s.d.'s in distances, angles and torsion angles; correlations between e.s.d.'s in cell parameters are only used when they are defined by crystal symmetry. An approximate (isotropic) treatment of cell e.s.d.'s is used for estimating e.s.d.'s involving l.s. planes.
Refinement. Refinement of *F*^2^ against ALL reflections. The weighted *R*-factor *wR* and goodness of fit *S* are based on *F*^2^, conventional *R*-factors *R* are based on *F*, with *F* set to zero for negative *F*^2^. The threshold expression of *F*^2^ > σ(*F*^2^) is used only for calculating *R*-factors(gt) *etc*. and is not relevant to the choice of reflections for refinement. *R*-factors based on *F*^2^ are statistically about twice as large as those based on *F*, and *R*- factors based on ALL data will be even larger.

### Fractional atomic coordinates and isotropic or equivalent isotropic displacement parameters (Å^2^)

**Table:**

	*x*	*y*	*z*	*U*_iso_*/*U*_eq_	Occ. (<1)
C25	1.0692 (2)	0.6151 (7)	−0.3351 (7)	0.150 (2)	
H25A	1.0867	0.6580	−0.2543	0.179*	
H25B	1.0716	0.5093	−0.3241	0.179*	
C24	1.0184 (2)	0.6518 (7)	−0.3292 (7)	0.153 (2)	
H24A	1.0162	0.7579	−0.3381	0.184*	
H24B	1.0012	0.6110	−0.4113	0.184*	
C2	0.31670 (15)	0.6261 (4)	0.2849 (4)	0.0741 (11)	
C1	0.26593 (19)	0.6458 (7)	0.3228 (6)	0.0935 (13)	
C27	1.1456 (2)	0.6184 (9)	−0.4560 (9)	0.208 (4)	
H27A	1.1556	0.6545	−0.5417	0.312*	
H27B	1.1502	0.5138	−0.4514	0.312*	
H27C	1.1652	0.6643	−0.3816	0.312*	
C26	1.0960 (3)	0.6514 (10)	−0.4462 (8)	0.213 (4)	
H26A	1.0783	0.6082	−0.5265	0.256*	
H26B	1.0931	0.7571	−0.4572	0.256*	
F1	0.2363 (3)	0.694 (2)	0.2215 (10)	0.137 (6)	0.71 (4)
F2	0.2468 (5)	0.5236 (12)	0.361 (3)	0.159 (9)	0.71 (4)
F3	0.2620 (2)	0.739 (2)	0.4226 (19)	0.145 (8)	0.71 (4)
F1A	0.2625 (6)	0.621 (7)	0.450 (2)	0.154 (19)	0.29 (4)
F2A	0.2343 (8)	0.556 (5)	0.269 (5)	0.147 (17)	0.29 (4)
F3A	0.2475 (14)	0.766 (3)	0.303 (7)	0.18 (3)	0.29 (4)
O1	0.43973 (9)	0.6693 (2)	0.4333 (2)	0.0641 (7)	
O4	0.57932 (9)	0.6725 (3)	0.2856 (2)	0.0709 (7)	
C10	0.46005 (13)	0.5620 (3)	0.1853 (3)	0.0632 (10)	
H10	0.4667	0.5224	0.1011	0.076*	
C5	0.41071 (13)	0.5803 (3)	0.2142 (3)	0.0589 (9)	
O2	0.51778 (10)	0.6830 (3)	0.4977 (2)	0.0840 (8)	
C3	0.35544 (15)	0.6606 (4)	0.3744 (4)	0.0694 (10)	
H3	0.3503	0.7004	0.4597	0.083*	
C8	0.48791 (14)	0.6551 (3)	0.4068 (3)	0.0577 (9)	
C4	0.40217 (13)	0.6371 (3)	0.3396 (3)	0.0576 (9)	
C9	0.49690 (13)	0.5990 (3)	0.2735 (3)	0.0551 (8)	
O3	0.55682 (9)	0.4843 (3)	0.1529 (3)	0.0868 (9)	
C6	0.37051 (14)	0.5468 (4)	0.1238 (4)	0.0765 (11)	
H6	0.3755	0.5086	0.0378	0.092*	
C11	0.54679 (13)	0.5768 (4)	0.2315 (3)	0.0611 (9)	
C7	0.32407 (15)	0.5687 (4)	0.1581 (4)	0.0843 (12)	
H7	0.2973	0.5451	0.0965	0.101*	
C12	0.62703 (14)	0.6568 (4)	0.2462 (4)	0.0668 (10)	
C17	0.64238 (15)	0.7362 (4)	0.1429 (4)	0.0784 (11)	
H17	0.6206	0.8001	0.0944	0.094*	
C15	0.72109 (15)	0.6315 (5)	0.1777 (5)	0.0847 (12)	
C16	0.68960 (15)	0.7248 (4)	0.1077 (4)	0.0831 (12)	
H16	0.7000	0.7809	0.0360	0.100*	
O5	0.76854 (11)	0.6082 (4)	0.1512 (4)	0.1166 (11)	
C13	0.65845 (18)	0.5638 (5)	0.3158 (4)	0.0971 (14)	
H13	0.6480	0.5083	0.3878	0.117*	
C14	0.70536 (18)	0.5511 (6)	0.2811 (5)	0.1108 (16)	
H14	0.7269	0.4863	0.3292	0.133*	
C19	0.83692 (17)	0.6115 (6)	0.0247 (5)	0.1171 (17)	
H19A	0.8573	0.6386	0.1070	0.141*	
H19B	0.8350	0.5050	0.0225	0.141*	
C18	0.78703 (16)	0.6698 (5)	0.0363 (5)	0.1025 (15)	
H18A	0.7882	0.7760	0.0450	0.123*	
H18B	0.7659	0.6451	−0.0453	0.123*	
C20	0.86150 (18)	0.6598 (6)	−0.0933 (6)	0.1232 (18)	
H20A	0.8609	0.7665	−0.0952	0.148*	
H20B	0.8425	0.6257	−0.1754	0.148*	
C23	0.99056 (18)	0.6129 (7)	−0.2183 (6)	0.134 (2)	
H23A	1.0081	0.6518	−0.1357	0.161*	
H23B	0.9920	0.5066	−0.2107	0.161*	
C21	0.91271 (18)	0.6116 (7)	−0.1003 (6)	0.1294 (19)	
H21A	0.9313	0.6457	−0.0174	0.155*	
H21B	0.9128	0.5050	−0.0969	0.155*	
C22	0.9393 (2)	0.6543 (7)	−0.2135 (6)	0.137 (2)	
H22A	0.9377	0.7607	−0.2197	0.165*	
H22B	0.9216	0.6160	−0.2960	0.165*	

### Atomic displacement parameters (Å^2^)

**Table:**

	*U*^11^	*U*^22^	*U*^33^	*U*^12^	*U*^13^	*U*^23^
C25	0.088 (4)	0.175 (6)	0.191 (6)	0.014 (4)	0.044 (4)	0.019 (5)
C24	0.103 (4)	0.190 (6)	0.172 (6)	0.028 (4)	0.051 (4)	0.016 (5)
C2	0.076 (3)	0.068 (2)	0.081 (3)	0.002 (2)	0.025 (2)	0.011 (2)
C1	0.090 (4)	0.099 (4)	0.095 (4)	0.004 (3)	0.026 (3)	0.011 (3)
C27	0.108 (5)	0.243 (9)	0.282 (10)	0.012 (5)	0.066 (6)	0.009 (8)
C26	0.137 (7)	0.279 (10)	0.235 (9)	0.037 (6)	0.088 (6)	0.023 (8)
F1	0.097 (4)	0.197 (15)	0.118 (6)	0.045 (6)	0.021 (4)	0.027 (6)
F2	0.103 (8)	0.120 (6)	0.26 (2)	0.014 (5)	0.083 (12)	0.075 (10)
F3	0.094 (4)	0.190 (15)	0.158 (11)	0.008 (6)	0.043 (5)	−0.069 (11)
F1A	0.106 (11)	0.25 (5)	0.117 (13)	0.009 (18)	0.061 (9)	0.032 (18)
F2A	0.083 (10)	0.15 (3)	0.21 (3)	−0.011 (12)	0.038 (14)	−0.03 (2)
F3A	0.18 (2)	0.12 (2)	0.27 (6)	0.079 (18)	0.10 (4)	0.07 (3)
O1	0.0811 (17)	0.0674 (15)	0.0456 (13)	0.0026 (12)	0.0160 (12)	−0.0070 (11)
O4	0.0756 (17)	0.0752 (16)	0.0639 (15)	−0.0077 (13)	0.0181 (12)	−0.0240 (13)
C10	0.083 (3)	0.067 (2)	0.0423 (19)	−0.0066 (18)	0.0218 (19)	−0.0059 (16)
C5	0.072 (2)	0.059 (2)	0.0467 (19)	−0.0125 (17)	0.0157 (18)	−0.0017 (15)
O2	0.089 (2)	0.115 (2)	0.0480 (14)	0.0021 (16)	0.0037 (14)	−0.0223 (14)
C3	0.086 (3)	0.064 (2)	0.061 (2)	0.004 (2)	0.025 (2)	0.0030 (17)
C8	0.078 (3)	0.0537 (19)	0.0421 (19)	0.0018 (17)	0.0112 (18)	−0.0019 (15)
C4	0.076 (2)	0.0484 (18)	0.050 (2)	−0.0019 (16)	0.0143 (18)	0.0045 (15)
C9	0.077 (2)	0.0517 (18)	0.0377 (18)	−0.0050 (16)	0.0124 (17)	−0.0009 (14)
O3	0.0876 (19)	0.0923 (19)	0.0828 (18)	−0.0046 (15)	0.0212 (15)	−0.0405 (15)
C6	0.081 (3)	0.091 (3)	0.060 (2)	−0.017 (2)	0.019 (2)	−0.006 (2)
C11	0.080 (3)	0.062 (2)	0.0414 (18)	−0.0022 (19)	0.0086 (17)	−0.0041 (16)
C7	0.078 (3)	0.102 (3)	0.074 (3)	−0.013 (2)	0.011 (2)	−0.001 (2)
C12	0.071 (2)	0.070 (2)	0.059 (2)	0.002 (2)	0.0062 (19)	−0.0184 (19)
C17	0.078 (3)	0.072 (2)	0.086 (3)	0.002 (2)	0.011 (2)	0.004 (2)
C15	0.069 (3)	0.096 (3)	0.090 (3)	0.002 (2)	0.012 (2)	−0.013 (3)
C16	0.070 (3)	0.081 (3)	0.100 (3)	−0.005 (2)	0.017 (2)	0.005 (2)
O5	0.076 (2)	0.146 (3)	0.129 (3)	0.0163 (19)	0.013 (2)	0.006 (2)
C13	0.105 (4)	0.118 (4)	0.070 (3)	0.015 (3)	0.019 (2)	0.016 (3)
C14	0.094 (4)	0.144 (4)	0.096 (3)	0.037 (3)	0.011 (3)	0.018 (3)
C19	0.067 (3)	0.150 (5)	0.135 (4)	0.004 (3)	0.016 (3)	0.000 (4)
C18	0.077 (3)	0.105 (3)	0.128 (4)	−0.007 (3)	0.026 (3)	−0.015 (3)
C20	0.085 (3)	0.138 (4)	0.151 (5)	0.009 (3)	0.035 (3)	−0.007 (4)
C23	0.077 (3)	0.161 (5)	0.166 (5)	0.012 (3)	0.028 (3)	0.015 (4)
C21	0.076 (3)	0.160 (5)	0.154 (5)	0.011 (3)	0.022 (3)	0.013 (4)
C22	0.091 (4)	0.171 (5)	0.154 (5)	0.020 (4)	0.036 (4)	0.003 (4)

### Geometric parameters (Å, º)

**Table:**

C25—C26	1.429 (8)	C9—C11	1.499 (5)
C25—C24	1.460 (8)	O3—C11	1.212 (4)
C25—H25A	0.9900	C6—C7	1.381 (5)
C25—H25B	0.9900	C6—H6	0.9500
C24—C23	1.451 (7)	C7—H7	0.9500
C24—H24A	0.9900	C12—C17	1.365 (5)
C24—H24B	0.9900	C12—C13	1.375 (5)
C2—C3	1.379 (5)	C17—C16	1.394 (5)
C2—C7	1.404 (5)	C17—H17	0.9500
C2—C1	1.506 (6)	C15—C14	1.375 (6)
C1—F3A	1.234 (17)	C15—C16	1.380 (6)
C1—F1A	1.300 (18)	C15—O5	1.387 (5)
C1—F2A	1.30 (2)	C16—H16	0.9500
C1—F2	1.323 (11)	O5—C18	1.417 (5)
C1—F1	1.329 (9)	C13—C14	1.385 (6)
C1—F3	1.332 (9)	C13—H13	0.9500
C27—C26	1.426 (9)	C14—H14	0.9500
C27—H27A	0.9800	C19—C20	1.481 (7)
C27—H27B	0.9800	C19—C18	1.505 (6)
C27—H27C	0.9800	C19—H19A	0.9900
C26—H26A	0.9900	C19—H19B	0.9900
C26—H26B	0.9900	C18—H18A	0.9900
O1—C4	1.376 (4)	C18—H18B	0.9900
O1—C8	1.396 (4)	C20—C21	1.502 (7)
O4—C11	1.349 (4)	C20—H20A	0.9900
O4—C12	1.425 (4)	C20—H20B	0.9900
C10—C9	1.339 (5)	C23—C22	1.482 (7)
C10—C5	1.438 (5)	C23—H23A	0.9900
C10—H10	0.9500	C23—H23B	0.9900
C5—C4	1.397 (4)	C21—C22	1.458 (7)
C5—C6	1.412 (5)	C21—H21A	0.9900
O2—C8	1.206 (4)	C21—H21B	0.9900
C3—C4	1.392 (5)	C22—H22A	0.9900
C3—H3	0.9500	C22—H22B	0.9900
C8—C9	1.469 (4)		
			
C26—C25—C24	123.3 (7)	C10—C9—C11	117.2 (3)
C26—C25—H25A	106.5	C8—C9—C11	122.3 (3)
C24—C25—H25A	106.5	C7—C6—C5	121.2 (4)
C26—C25—H25B	106.5	C7—C6—H6	119.4
C24—C25—H25B	106.5	C5—C6—H6	119.4
H25A—C25—H25B	106.5	O3—C11—O4	122.8 (3)
C23—C24—C25	123.7 (6)	O3—C11—C9	123.3 (3)
C23—C24—H24A	106.4	O4—C11—C9	113.9 (3)
C25—C24—H24A	106.4	C6—C7—C2	119.4 (4)
C23—C24—H24B	106.4	C6—C7—H7	120.3
C25—C24—H24B	106.4	C2—C7—H7	120.3
H24A—C24—H24B	106.5	C17—C12—C13	119.6 (4)
C3—C2—C7	120.4 (4)	C17—C12—O4	120.8 (3)
C3—C2—C1	120.6 (4)	C13—C12—O4	119.6 (4)
C7—C2—C1	119.0 (4)	C12—C17—C16	120.8 (4)
F3A—C1—F1A	104.7 (15)	C12—C17—H17	119.6
F3A—C1—F2A	104.9 (19)	C16—C17—H17	119.6
F1A—C1—F2A	100.8 (14)	C14—C15—C16	119.4 (4)
F3A—C1—F2	130.3 (12)	C14—C15—O5	115.4 (4)
F1A—C1—F2	60.1 (18)	C16—C15—O5	125.2 (4)
F2A—C1—F2	44.9 (18)	C15—C16—C17	119.5 (4)
F3A—C1—F1	50 (3)	C15—C16—H16	120.2
F1A—C1—F1	135.3 (10)	C17—C16—H16	120.2
F2A—C1—F1	63 (2)	C15—O5—C18	120.6 (4)
F2—C1—F1	105.6 (10)	C12—C13—C14	120.1 (4)
F3A—C1—F3	58 (3)	C12—C13—H13	120.0
F1A—C1—F3	51 (2)	C14—C13—H13	120.0
F2A—C1—F3	129.4 (10)	C15—C14—C13	120.6 (4)
F2—C1—F3	106.4 (8)	C15—C14—H14	119.7
F1—C1—F3	105.3 (7)	C13—C14—H14	119.7
F3A—C1—C2	117.1 (10)	C20—C19—C18	116.3 (5)
F1A—C1—C2	111.8 (10)	C20—C19—H19A	108.2
F2A—C1—C2	115.8 (9)	C18—C19—H19A	108.2
F2—C1—C2	112.2 (6)	C20—C19—H19B	108.2
F1—C1—C2	112.7 (5)	C18—C19—H19B	108.2
F3—C1—C2	113.9 (6)	H19A—C19—H19B	107.4
C26—C27—H27A	109.5	O5—C18—C19	108.5 (4)
C26—C27—H27B	109.5	O5—C18—H18A	110.0
H27A—C27—H27B	109.5	C19—C18—H18A	110.0
C26—C27—H27C	109.5	O5—C18—H18B	110.0
H27A—C27—H27C	109.5	C19—C18—H18B	110.0
H27B—C27—H27C	109.5	H18A—C18—H18B	108.4
C27—C26—C25	125.2 (8)	C19—C20—C21	116.8 (5)
C27—C26—H26A	106.0	C19—C20—H20A	108.1
C25—C26—H26A	106.0	C21—C20—H20A	108.1
C27—C26—H26B	106.0	C19—C20—H20B	108.1
C25—C26—H26B	106.0	C21—C20—H20B	108.1
H26A—C26—H26B	106.3	H20A—C20—H20B	107.3
C4—O1—C8	122.6 (3)	C24—C23—C22	122.6 (5)
C11—O4—C12	115.6 (3)	C24—C23—H23A	106.7
C9—C10—C5	122.1 (3)	C22—C23—H23A	106.7
C9—C10—H10	119.0	C24—C23—H23B	106.7
C5—C10—H10	119.0	C22—C23—H23B	106.7
C4—C5—C6	118.0 (3)	H23A—C23—H23B	106.6
C4—C5—C10	117.5 (3)	C22—C21—C20	120.2 (5)
C6—C5—C10	124.5 (3)	C22—C21—H21A	107.3
C2—C3—C4	119.9 (3)	C20—C21—H21A	107.3
C2—C3—H3	120.0	C22—C21—H21B	107.3
C4—C3—H3	120.0	C20—C21—H21B	107.3
O2—C8—O1	116.8 (3)	H21A—C21—H21B	106.9
O2—C8—C9	126.7 (3)	C21—C22—C23	120.6 (5)
O1—C8—C9	116.4 (3)	C21—C22—H22A	107.2
O1—C4—C3	118.0 (3)	C23—C22—H22A	107.2
O1—C4—C5	120.9 (3)	C21—C22—H22B	107.2
C3—C4—C5	121.1 (4)	C23—C22—H22B	107.2
C10—C9—C8	120.4 (3)	H22A—C22—H22B	106.8

### Hydrogen-bond geometry (Å, º)

Cg1 and Cg2 are the centroids of the C2–C7 and C12–C17 rings respectively.

**Table:**

*D*—H···*A*	*D*—H	H···*A*	*D*···*A*	*D*—H···*A*
C10—H10···O3^i^	0.95	2.56	3.396 (5)	146
C27—H27*C*···F1^ii^	0.98	2.52	3.432 (13)	154
C6—H6···*Cg*2^i^	0.95	3.09	3.899	144
C16—H16···*Cg*2^iii^	0.95	3.33	4.120	142
C3—H3···*Cg*1^iv^	0.95	3.43	4.325	163

Symmetry codes: (i) −*x*+1, −*y*+1, −*z*; (ii) *x*+1, −*y*+3/2, *z*−1/2; (iii) *x*, −*y*+3/2, *z*−1/2; (iv) *x*, −*y*+3/2, *z*+1/2.

## References

[bb1] Bernstein, J., Davis, R. E., Shimoni, L. & Chang, N.-L. (1995). *Angew. Chem. Int. Ed. Engl.* **34**, 1555–1573.

[bb2] Borges, F., Roleira, F., Milhazes, N., Santana, L. & Uriarte, E. (2005). *Curr. Med. Chem.* **12**, 887–916. 10.2174/0929867053507315 15853704

[bb3] Bruker (2009). *APEX2*, *SADABS*, *SAINT-Plus* and *XPREP* Bruker AXS Inc., Madison, Wisconsin, USA.

[bb4] Farrugia, L. J. (2012). *J. Appl. Cryst.* **45**, 849–854.

[bb5] Hejchman, E. B., Konc, J. T., Maciejewska, D. & Kruszewska, H. (2011). *Synth. Commun.* **41**, 2392–2402.

[bb6] Kontogiorgis, C. A. & Hadjipavlou-Litina, D. J. (2005). *J. Med. Chem.* **48**, 6400–6408. 10.1021/jm0580149 16190766

[bb7] Macrae, C. F., Bruno, I. J., Chisholm, J. A., Edgington, P. R., McCabe, P., Pidcock, E., Rodriguez-Monge, L., Taylor, R., van de Streek, J. & Wood, P. A. (2008). *J. Appl. Cryst.* **41**, 466–470.

[bb8] Mahadevan, K. M., Harish Kumar, H. N., Masagalli, J. N. & Srinivasa, H. T. (2013). *Mol. Cryst. Liq. Cryst.* **570**, 20–35.

[bb9] Palakshamurthy, B. S., Sreenivasa, S., Srinivasa, H. T., Roopashree, K. R. & Devarajegowda, H. C. (2013). *Acta Cryst.* E**69**, o212. 10.1107/S1600536813000214 PMC3569748 23424494

[bb10] Sheldrick, G. M. (2007). *SADABS* University of Göttingen, Germany.

[bb11] Sheldrick, G. M. (2008). *Acta Cryst.* A**64**, 112–122. 10.1107/S0108767307043930 18156677

